# Supplementary material for: Venomix: a simple bioinformatic pipeline for identifying and characterizing toxin gene candidates from transcriptomic data
Source: PeerJ. 2018 Jul 31;6:e5361. doi: 10.7717/peerj.5361 (PMC6074769; doi:10.7717/peerj.5361)
Supplement: Supplemental Information 3 [file peerj-06-5361-s003.gz › FinalOutput_E-20/Snaclec_lebecin_subunit_alpha_9/finaltree.pdf]

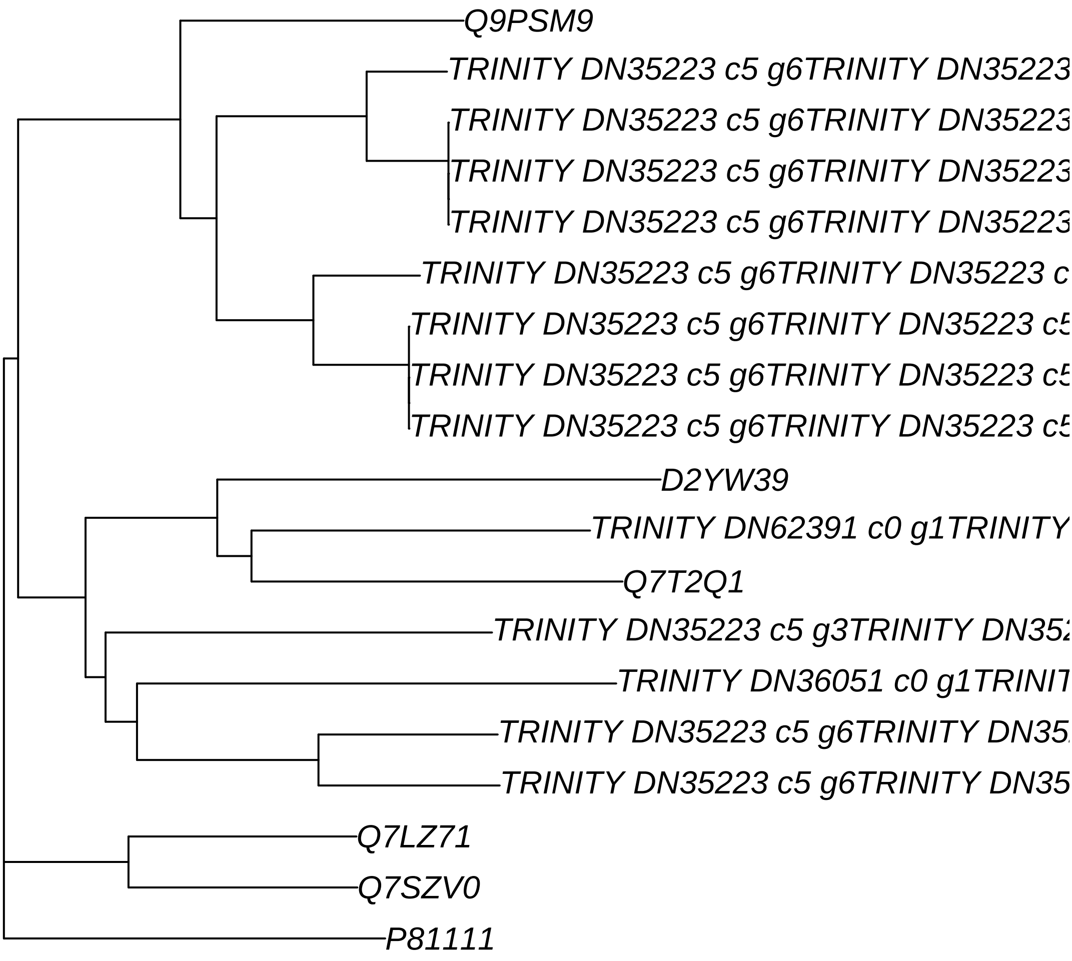
Q9PSM9
TRINITY DN35223 c5 g6TRINITY DN35223
TRINITY DN35223 c5 g6TRINITY DN35223
TRINITY DN35223 c5 g6TRINITY DN35223
TRINITY DN35223 c5 g6TRINITY DN35223
TRINITY DN35223 c5 g6TRINITY DN35223 c
TRINITY DN35223 c5 g6TRINITY DN35223 c5
TRINITY DN35223 c5 g6TRINITY DN35223 c5
TRINITY DN35223 c5 g6TRINITY DN35223 c5
D2YW39
TRINITY DN62391 c0 g1TRINITY
Q7T2Q1
TRINITY DN35223 c5 g3TRINITY DN352
TRINITY DN36051 c0 g1TRINIT
TRINITY DN35223 c5 g6TRINITY DN35
TRINITY DN35223 c5 g6TRINITY DN35
Q7LZ71
Q7SZV0
P81111
